# Novel lncRNA *UGGT1-AS1* Regulates *UGGT1* Expression in Breast Cancer Cell Line

**DOI:** 10.3390/ijms26115108

**Published:** 2025-05-26

**Authors:** Klaudia Samorowska, Elżbieta Wanowska, Michał Wojciech Szcześniak

**Affiliations:** Faculty of Biology, Institute of Human Biology and Evolution, Adam Mickiewicz University in Poznan, Uniwersytetu Poznańskiego 6, 61-614 Poznań, Poland

**Keywords:** natural antisense transcript, A-to-I RNA editing, breast cancer, lncRNA

## Abstract

Long non-coding RNAs (lncRNAs) are transcripts over 200 nucleotides long that do not encode proteins. Although many lncRNAs remain uncharacterized, they are known to play diverse regulatory roles in gene expression. A group of lncRNAs called natural antisense transcripts can form double-stranded structures with their sense partners due to sequence complementarity. These duplexes can become substrates for A-to-I RNA editing, an epitranscriptomic modification mediated by ADAR enzymes. RNA editing is known to influence transcript splicing, affect the resulting gene expression product or alter RNA stability, all of which can impact cancer cell biology. Here, we show a novel natural antisense transcript, *UGGT1-AS1*, that we have identified and characterized in terms of its cellular localization and sense partner interactions. Furthermore, we demonstrate that *UGGT1-AS1* affects cell proliferation and regulates the stability of the *UGGT1* sense transcript. Finally, using publicly available RNA sequencing data, we identify A-to-I RNA editing events in the protein-coding gene *UGGT1* and further confirm them by RT-PCR and Sanger sequencing in MCF7 cell lines. We hypothesize that *UGGT1-AS1* may act as a triggering factor for the A-to-I RNA editing process in its sense partner. Our findings highlight the regulatory role of *UGGT1-AS1* and suggest its involvement in RNA editing and cancer biology.

## 1. Introduction

Long non-coding RNAs (lncRNAs) are defined as transcripts >200 nucleotides long that do not encode functional proteins. They have already been linked to virtually all biological processes in the cell and are also associated with human diseases, including cancers [1,2]. The functions played by lncRNAs have diverse molecular backgrounds, which underlies the difficulty in the functional study of these molecules, especially if the targeted genes are unknown. Many lncRNAs share similar features with mRNAs: they are transcribed by RNA polymerase II (RNAPII), spliced and polyadenylated. They can bind an extensive range of molecules and control gene expression at multiple levels. Through interactions with DNA, RNA and proteins, they are able to affect chromatin structure, transcription, alternative splicing, the stability of other RNA molecules and translation [3,4]. A fraction of lncRNAs, called natural antisense transcripts (NATs), are expressed from DNA strands opposite to another gene, typically a protein-coding gene, referred to as a sense partner. One of the most important functions of NATs is their ability to form RNA:RNA duplexes [5]. Double-stranded RNAs may function as substrates for adenosine deaminases acting on RNA (ADAR) enzymes that conduct the adenosine to inosine (A-to-I) RNA editing process, i.e., adenosines are deaminated to inosines [6,7]. A-to-I editing affects various aspects of RNA life, such as the pattern of alternative splicing, protein coding capacity and RNA stability, and also change their subcellular localization, sometimes with a remarkable effect on cell biology [8]. For instance, in mRNAs of the chloride channel Gabra3, isoleucine is substituted for methionine in a subunit of the GABAA receptor. While Gabra3 overexpression promotes breast cancer cell invasion, migration and metastasis, the edited Gabra3 has the opposite effect [9]. Of note, it is estimated that 95% of nascent pre-mRNAs are subject to this type of modification [10].

The ADAR-mediated process of A-to-I RNA editing plays a significant role in cancer progression [11,12,13]. The implications of deregulated A-to-I RNA editing in cancers are profound and complex. The upregulation of RNA editing can result from the increased expression of ADAR genes, as seen in breast cancer cells, particularly at 3′ UTR sites. Conversely, downregulation may occur due to the decreased expression or loss of ADAR enzymes, which is observed, for example, in gastric cancer. Research suggests that a high level of RNA editing can either boost or decrease tumor aggressiveness [14,15].

In this research, we focus on A-to-I RNA editing events occurring on *UGGT1* gene transcripts. *UGGT1* encodes UDP-glucose glycoprotein glucosyltransferase 1, a protein essential for maintaining proper glycoprotein folding in the endoplasmic reticulum (ER) [16]. Recent studies have implicated *UGGT1* in cancer, particularly in low-grade gliomas (LGG), where increased *UGGT1* expression correlates with poor prognosis. Interestingly, specific A-to-I editing events on *UGGT1* transcripts, particularly at chr2:128952084 (hg19), are associated with elevated *UGGT1* expression levels [17]. This suggests that RNA editing may play a key role in regulating *UGGT1* function in cancer. Our study investigates the interaction between *UGGT1* and a newly identified natural antisense transcript (NAT) called *UGGT1-AS1*, which was found to be enriched in nuclear cell fractions. The *UGGT1*-*AS1* transcript appears to form RNA duplexes with *UGGT1*, potentially serving as a substrate for ADAR-mediated A-to-I RNA editing, a mechanism that may contribute to the regulation of *UGGT1* expression. Given the importance of RNA editing in gene regulation, our findings indicate that *UGGT1-AS1* may influence cancer progression through the modulation of *UGGT1* transcript stability and expression, providing new insights into the molecular mechanisms underlying cancer biology.

## 2. Results

### 2.1. A-to-I Editing Events in Breast Cancer

**Figure 1:**
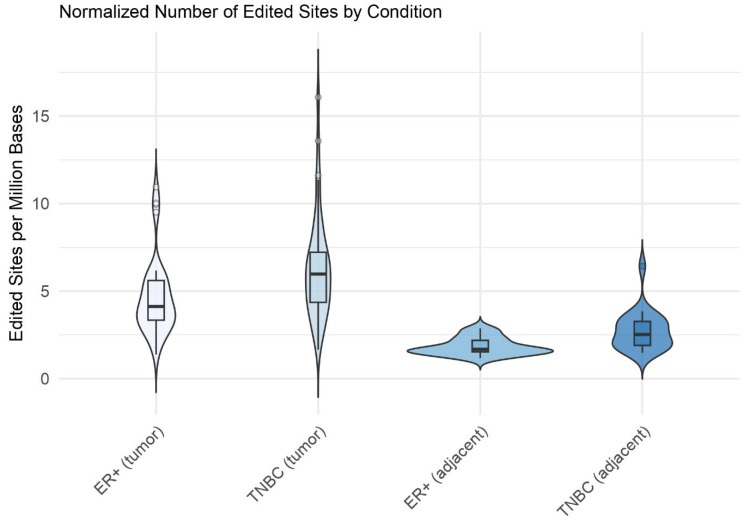
Comparison of the number of edited sites across different conditions. The graph shows the numbers of edited RNA sites detected in two breast cancer subtypes, ER+ breast cancer and triple-negative breast cancer (TNBC), comparing the tumor tissues to their adjacent non-cancerous tissues, normalized for sequencing depth, which indicates that observed differences were not artifacts from sequencing depth, ensuring comparability between samples. The graph represents statistically significant differences in the number of edited sites between the cancerous and non-cancerous samples.

RNA editing sites were identified with SPRINT, using RNA-Seq reads as an input: 42 ER+ (Estrogen Receptor—positive) samples, 30 samples from healthy tissue adjacent to ER+ tumors, 42 TNBC (triple-negative breast cancer) samples and 21 samples from healthy tissue adjacent to TNBC tumors. The number of edited sites ranged from 5609 in the uninvolved TNBC sample SRR1313174 to 106,252 in the TNBC tumor sample SRR1313146. Notably, the cancerous samples displayed far more edited sites than the non-cancerous samples (Figure 1). These differences were statistically significant, as shown by the Mann–Whitney *p*-values for TNBC (tumor) vs. TNBC (adjacent) (*p* = 7.97 × 10^−8^) and ER+ (tumor) vs. ER+ (adjacent) (*p* = 1.61 × 10^−12^).

To identify the RNA editing events that best distinguished the tumor samples (ER+, TNBC) from their adjacent, non-cancerous counterparts, we first filtered the sites to include only those detected in at least 40% of either the control samples or the corresponding cancer samples. For each remaining site, we calculated the ratio of its prevalence (i.e., percentage of samples with given editing event detected) in the breast cancer samples to its prevalence in the control samples. This ratio enabled us to assess how the two groups differed in terms of the presence or absence of each editing event. We then computed Z-scores for these ratios and retained only those sites showing the largest differences between groups (|Z-score| > 1.96). Finally, we further filtered these sites to highlight only those overlapped by natural antisense transcripts, obtaining 22 top events for the ER+ samples and 39 for the TNBC samples, with a single common event located on chromosome 7 at position 77,056,248, overlapping SPDYE18 (Speedy/RINGO Cell Cycle Regulator Family Member E18). Additional genes identified included *GALNT10*, *NPIPB4*, *POGZ*, *MED13L* and *UGGT1*, most of which had confirmed roles in cancers. Of these, *UGGT1* (UDP-glucose glycoprotein glucosyltransferase) was selected for further investigation because the A-to-I editing of its transcripts is associated with increased expression levels, which, according to the literature, correlates with poor prognosis in cancer patients [16,17,18].

### 2.2. UGGT1-AS1 Is a Natural Antisense Transcript of the UGGT1 Gene

**Figure 2:**
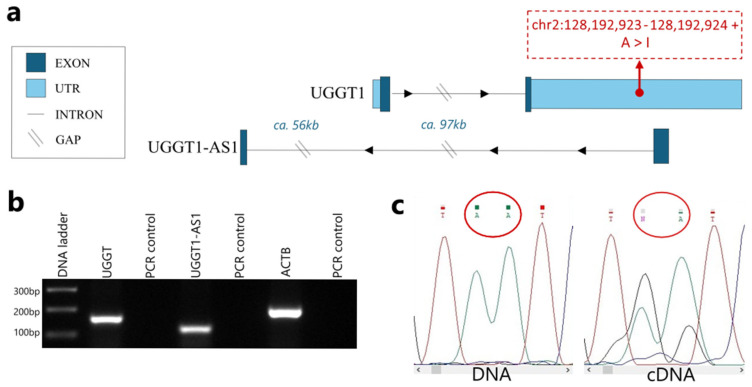
The natural antisense transcript of the *UGGT1* gene. (**a**) Genomic context of the sense (*UGGT1*) and antisense (*UGGT1*-*AS1*) transcripts. Dark blue: exons; light blue: untranslated regions; thin black lines: introns (diagonal lines denote gaps in the intronic sequence, used to simplify the graph. (**b**) Results of RT-PCR on the MCF7 cells material, showing that *UGGT1* and *UGGT1*-*AS1* are expressed. (**c**) Results of *UGGT1* Sanger sequencing, visualized in Chromas viewer. DNA and RNA were isolated from the same MCF7 cells. RNA was reverse transcribed to cDNA and both acids were used in the PCRs. Two loci predicted to be edited in breast cancer were checked, and the predictions of A-to-I RNA editing events were confirmed in vitro.

It is well known that approximately 70% of mammalian genes have their own natural antisense transcripts [19,20]. While *UGGT1* does not have an annotated natural antisense transcript in databases (ENSEMBL, RefSeq), our custom assembly of the human transcriptome revealed a novel NAT, which we dubbed *UGGT1-AS1* (UGGT1 Antisense 1) [21]. Figure 2a shows the genomic context of both *UGGT1* and *UGGT1-AS1*, along with the localization of A-to-I RNA editing events. This simplified graph provides a clear overview of the relative positions of the two genes. From the two breast cancer subtypes analyzed in silico, we chose the ER+ subtype, represented by the MCF7 cell line, for further in vitro experiments. The expression of both *UGGT1* and *UGGT1*-*AS1* was confirmed in the MCF7 breast cancer cell line in vitro using a PCR (Figure 2b). In order to check if the predicted editing events occurred in transcripts of *UGGT1* gene, a RT-PCR followed by Sanger sequencing was performed. The experiment confirmed that RNA editing took place in two distinct loci (Figure 2c).

### 2.3. UGGT1-AS1 Transcripts Are Located in the Cell Nucleus

**Figure 3:**
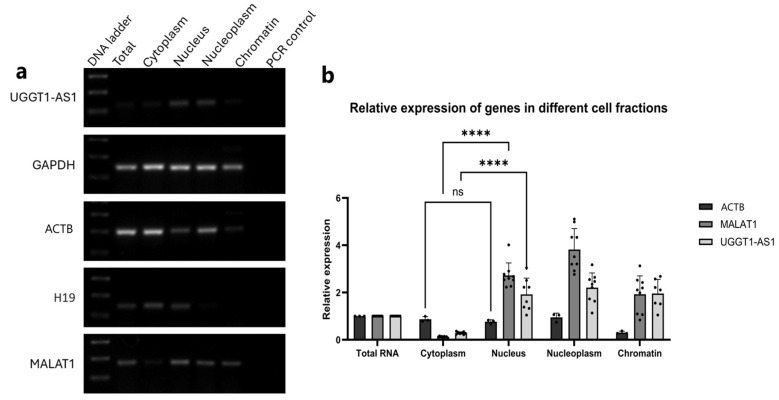
Subcellular localization of UGGT1-AS1 and its protein interactome. (**a**) RT-PCR results showing UGGT1-AS1 expression in different cell fractions. *MALAT1* and *ACTB* were used as markers for the chromatin and cytoplasmic fractions, respectively. *GAPDH* was used as a reference gene in reverse transcription. (**b**) RT-qPCR results showing the relative expression of the *UGGT1-AS1* gene with two control genes, *ACTB* and *MALAT1*. The expression of the genes was normalized to the total cell fraction and to the *GAPDH* reference gene expression (**** *p* < 0.0001, ns not significant).

One of the key indicators of a transcript’s function is its cellular localization. Since the process of A-to-I RNA editing takes place cotranscriptionally in the cell nucleus [22], it was essential to determine the cellular localization of the *UGGT1-AS1* transcripts. Both the RT-PCR and RT-qPCR experiments showed that *UGGT1-AS1* was enriched in the nuclear cell fractions, including the nucleus, nucleoplasm and chromatin, with a significantly lower distribution in the cytoplasmic fraction (Figure 3a,b).

### 2.4. Cellular Roles for UGGT1-AS1

*UGGT1-AS1* positively regulates *UGGT1* expression and affects cells proliferation.

**Figure 4:**
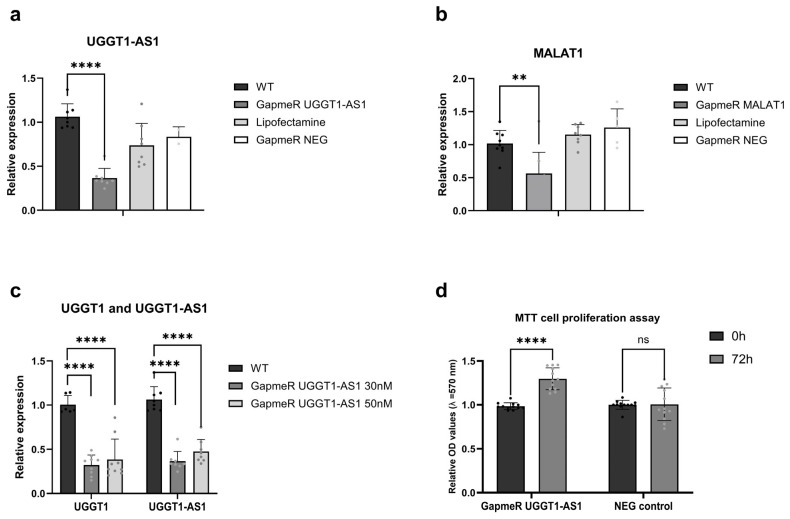
*UGGT1-AS1* regulates MCF7 cell proliferation and its sense partner expression. (**a**) Relative expression of the *UGGT-AS1* gene after transfection with GapmeRs. The transfection was performed using a lipofectamine reagent, which alone also served as a negative control. The second negative control was carried out with custom negative control GapmeRs (NEG control). GapmeR *UGGT1*-*AS1*—cells transfected with gapmeR against *UGGT1-AS1*. (**b**) Relative expression of MALAT1 gene after GapmeR treatment. The knock-down of *MALAT1* was used as a positive control for GapmeRs. (**c**) Impact of *UGGT1-AS1* silencing on the expression levels of the *UGGT1* gene. The silencing of the *UGGT1-AS1* gene with GapmeRs was performed at two different concentrations (30 nM and 50 nM). The experiments were conducted with three biological and three technical replicates. (**d**) MCF7 cell proliferation after *UGGT1-AS1* GapmeR treatment. Absorbance was measured at OD = 570 nm in the MCF7 cells after the *UGGT-AS1* and negative control (NEG) GapmeR treatments. An increase in cell proliferation was observed at 72 h post-treatment in the *UGGT1-AS1* knock-down group (** *p* < 0.01; **** *p* < 0.0001).

To obtain hints as to potential functionalities of *UGGT-AS1* and, possibly, its impact on *UGGT1*, we performed a loss of function experiment with GapmeRs. We used antisense LNA GapmeRs to knock down *UGGT1-AS1* in the MCF7 cell line. Figure 4a shows a significant reduction in *UGGT1-AS1’s* relative expression after transfection with GapmeRs, confirming the efficiency of the knock-down. The negative controls included cells treated with lipofectamine alone and custom negative control GapmeRs (NEG control), which did not show a substantial decrease in *UGGT1-AS1* levels, validating the specificity of the used GapmeRs.

We also included *MALAT1* knock-down as a positive control. As shown in Figure 4b, *MALAT1* expression was significantly reduced by its specific GapmeRs, confirming the reliability of the knock-down strategy. The relative expression of *MALAT1* dropped compared to its wild type (WT) levels in the presence of *MALAT1*-specific GapmeRs, while the negative controls did not lead to such a reduction.

It is established that natural antisense transcripts can regulate the expression of their sense partners in two general manners, namely leading to concordant or discordant regulation [23]. This is why we decided to test the expression levels of *UGGT1* after silencing its natural antisense transcript. Figure 4c demonstrates that *UGGT1-AS1* knock-down with two different concentrations of GapmeRs (30 nM and 50 nM) resulted in altered *UGGT1* expression levels. In general, *UGGT1* expression levels decreased in response to *UGGT1-AS1* knock-down, suggesting a potential concordant regulatory relationship.

Our next goal was to look into the cellular roles of *UGGT1-AS1* in the biology of cancer cells. We decided to use the MTT assay and performed it on cells after transfection with GapmeRs to check the influence of *UGGT1-AS1* on cell proliferation. The results of our experiment (Figure 4d) show that the knock-down of *UGGT1-AS1* increased cell proliferation in MCF7 breast cancer cells compared to cells treated with negative control GapmeRs, suggesting that *UGGT1-AS1* may play an inhibitory role in cell proliferation in the studied breast cancer cell line.

*UGGT1*-*AS1* increases *UGGT1* stability by forming an RNA:RNA duplex.

**Figure 5:**
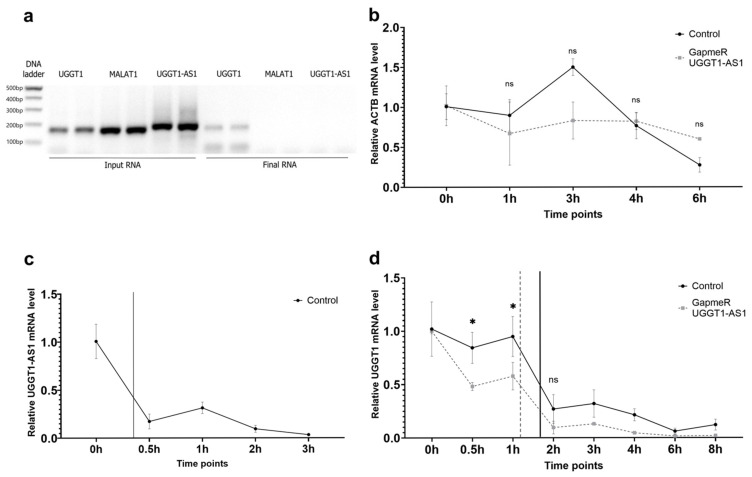
*UGGT1* and *UGGT1-AS1* form a duplex that increases sense gene stability. (**a**) Electrophoresis gel of the *UGGT1, UGGT1-AS1* and *MALAT1* genes after the RAP-RNA experiment. RNA probes (biotinylated antisense oligos) were designed to hybridize with *UGGT1-AS1*. The presence of a sense transcript in the last stage of the experiment (final RNA) confirms RNA:RNA interactions between the sense and antisense transcripts. (**b**) Relative expression of the *ACTB* control gene after actinomycin D treatment, demonstrating no effect of *UGGT1-AS1* silencing on *ACTB* stability. (**c**) Relative expression of the *UGGT1-AS1* gene after actinomycin D treatment. The vertical line shows the half-lives of the *UGGT1-AS1* transcripts. (**d**) Relative expression of the *UGGT1* gene after actinomycin D treatment, indicating a significant decreased stability of the *UGGT1* transcripts after *UGGT1*-*AS1* knock-down. The vertical lines represent the half-lives of the *UGGT1* transcripts in two conditions (ns not significant; * *p* < 0.05).

Given the presence of reverse complement nucleotides between *UGGT1-AS1* and *UGGT1*, we hypothesized that *UGGT1-AS1* and *UGGT1* might form an RNA duplex that would be a substrate for the ADAR enzyme, which would further trigger editing in *UGGT1* and increase the stability of its mRNAs. To check this hypothesis, we performed RNA antisense purification (RAP) using biotin-labeled DNA probes targeting *UGGT1*-AS1. We found that *UGGT1* mRNA was significantly increased in the biotin-labeled *UGGT1-AS1* pull-down samples compared to the negative control, confirming a direct interaction between *UGGT1* and *UGGT1-AS1* (Figure 5a). To evaluate the stability of the *UGGT1* and *UGGT1-AS1* transcripts, we used actinomycin D, a reagent known as the inhibitor of the transcription process by intercalation into DNA [24]. We wanted to check not only the general stability of these transcripts but also if there were changes in the half-life of the RNA before and after *UGGT1*-*AS1* knock-down. We tested the *UGGT1* stability by inhibiting RNA polymerase II transcription with actinomycin D (ActD) for 8 h both for cells treated with the GapmeR against *UGGT1-AS1* and the GapmeR control. The relative expression of the *ACTB* control gene, whose stability dropped similarly in samples with and without *UGGT1-AS1* GapmeR, is demonstrated in Figure 5b, confirming the effectiveness of the actinomycin D treatment and the reliability of our experiment. A reduction in *UGGT1-AS1* levels over an 8 h period post-treatment with actinomycin D is shown in Figure 5c, indicating its half-life to be relatively short. Figure 5d highlights the impact of *UGGT1-AS1* knock-down on *UGGT1* transcript stability. The data suggest that *UGGT1* mRNA levels decrease more rapidly when *UGGT1-AS1* is knocked down, implying that *UGGT1-AS1* might stabilize *UGGT1* transcripts under normal conditions. This observation supports the hypothesis that *UGGT1-AS1* positively regulates *UGGT1* mRNA stability, potentially through RNA interactions that protect *UGGT1* from degradation. Together, our findings show that *UGGT1-AS1* and *UGGT1* may create an RNA duplex to improve *UGGT1* mRNA stability.

## 3. Discussion

Antisense long non-coding RNAs (lncRNAs), also known as natural antisense transcripts (NATs), represent a significant and abundant fraction of lncRNAs. These transcripts are transcribed from the opposite DNA strand of a corresponding gene, which is typically a protein-coding gene referred to as the sense partner [25,26]. Antisense transcription is widespread in the genome, with studies estimating that up to 70% of genes exhibit antisense transcription [19,20].

The functional significance of NATs lies in their ability to regulate gene expression and RNA processing through multiple mechanisms [27,28]. One of the best-characterized mechanisms involves the recruitment of the Polycomb Repressive Complex 2 (PRC2), a gene-silencing complex that plays a critical role in maintaining transcriptional repression. PRC2 exerts its repressive function by catalyzing the trimethylation of lysine 27 on histone 3 (H3K27me3), a well-known epigenetic modification associated with transcriptionally inactive genomic regions. This regulatory mechanism represents an in cis effect, meaning that the antisense transcript influences the expression of its neighboring sense gene. Moreover, this effect is an example of discordant regulation, where an increase in the expression of the NAT leads to a corresponding inhibition of its sense counterpart. In addition to epigenetic silencing, NATs can regulate gene expression through RNA:RNA base pairing interactions. This direct hybridization allows NATs to impact various aspects of RNA processing, including pre-mRNA splicing, stability and translation. One common mode of action involves masking critical splice sites or other regulatory sequences, thereby interfering with normal pre-mRNA splicing events [29].

Another crucial function of NATs is their ability to form double-stranded RNA (dsRNA) structures with their sense transcripts, which can trigger RNA editing mechanisms. A particularly well-studied example of this phenomenon is adenosine-to-inosine (A-to-I) RNA editing, a process catalyzed by adenosine deaminases acting on RNA (ADAR enzymes) [30,31]. A-to-I editing can alter RNA stability, splicing and translation efficiency, thereby fine-tuning gene expression. For instance, Salameh et al. demonstrated that PCA3 lncRNA, a well-characterized tumor suppressor in prostate cancer, binds its sense partner PRUNE2, facilitating ADAR-mediated A-to-I RNA editing. This editing event ultimately leads to the suppression of PRUNE2 expression, highlighting the regulatory influence of NATs on oncogene or tumor suppressor function [32].

In the present study, we identified a novel natural antisense transcript, *UGGT1-AS1*, which originates from the reverse strand of the *UGGT1* gene. To gain insight into its potential function, we characterized *UGGT1-AS1* in terms of its expression patterns and subcellular localization. Our analyses revealed that *UGGT1-AS1* is predominantly localized in the nucleus, suggesting a possible role in nuclear RNA processing or transcriptional regulation.

Given its nuclear localization and antisense orientation, we hypothesized that *UGGT1-AS1* could be involved in facilitating the A-to-I RNA editing of *UGGT1*. To test this hypothesis, we used RT-PCR followed by Sanger sequencing, which confirmed the presence of A-to-I editing events in its sense partner, *UGGT1*. However, it remained unclear whether this editing was directly mediated by a *UGGT1*-*AS1*:*UGGT1* RNA duplex, or if other factors were contributing to the observed modifications.

To explore this possibility further, we conducted an RAP-RNA assay, which confirmed a direct RNA:RNA interaction between *UGGT1* and *UGGT1-AS1*. This finding supports the idea that *UGGT1-AS1* may function as a scaffold for recruiting ADAR enzymes to its sense partner, thereby promoting targeted RNA editing events.

To determine the functional impact of *UGGT1-AS1* on *UGGT1* expression and RNA editing, we performed a knock-down experiment using LNA GapmeRs. Following the *UGGT1-AS1* knock-down, we observed a decrease in both the *UGGT1-AS1* and *UGGT1* expression levels. However, despite the overall reduction in expression levels, the proportion of A-to-I edited bases in *UGGT1* remained unchanged.

This observation suggests that RNA editing efficiency is dependent on the relative stoichiometry of the interacting RNAs, rather than their absolute expression levels. Even though *UGGT1* and *UGGT1-AS1* levels were both reduced, their ratio remained consistent, allowing for the same proportion of adenosine residues to be converted to inosine. These findings indirectly support the hypothesis that *UGGT1-AS1* facilitates the A-to-I RNA editing of *UGGT1*, possibly through direct RNA duplex formation.

The regulatory effects of *UGGT1-AS1* on *UGGT1* expression and processing have significant implications for cancer biology. Our study found that knocking down *UGGT1* led to the increased proliferation of MCF7 breast cancer cells, suggesting that *UGGT1* may function as a tumor suppressor gene. Given that *UGGT1-AS1* influences *UGGT1* expression and RNA processing, it emerges as a potentially valuable therapeutic target in breast cancer research.

Future studies are needed to further dissect the precise molecular mechanisms by which *UGGT1-AS1* regulates *UGGT1* stability and function. Investigating whether *UGGT1-AS1* influences additional RNA modifications, chromatin remodeling events or protein interactions will be essential for fully understanding its biological significance. Additionally, exploring the role of *UGGT1-AS1* in other cancer types could provide new insights into its broader relevance in tumor progression and metastasis. The validation of our results in other cellular models or in vivo could determine whether the observed regulatory effects are more widely applicable. Moreover, using more advanced techniques like CRISPR-based approaches could further define roles of *UGGT1-AS1* in carcinogenesis and various processes like drug resistance, cell migration or invasion.

## 4. Materials and Methods

### 4.1. Pre-Processing of RNA-Seq Data

The RNA-Seq data were downloaded from the Sequence Read Archive (SRA, ID: SRP042620). The dataset comprised 135 sequencing runs, representing four sample types: Estrogen Receptor-positive (ER+) breast cancer primary tumor samples (n = 42), uninvolved breast tissue adjacent to ER+ primary tumor (n = 30), triple-negative breast cancer (TNBC) primary tumor samples (n = 42) and uninvolved breast tissue adjacent to TNBC primary tumor (n = 21). The RNA-Seq reads were subjected to quality filtering, trimming and adapter removal using BBDuk2 version 37.02 from the BBTools package (Joint Genome Institute). The following settings were applied: forcetrimleft = 6; qtrim = w; trimq = 20; maq = 10; rref = bbmap/resources/adapters.fa; k = 23; mink = 11; hdist = 1 tbo; tpe; minlength = 50; and removeifeitherbad = t.

### 4.2. Search for Adenosine-to-Inosine Editing Sites

A bioinformatic prediction of A-to-I RNA editing events was conducted using a SPRINT toolkit version 0.1.7 [33] with the “bwa” and “samtools” parameters, mapping each sequencing run individually to the human reference genome (GRCh38). Repetitive sequences for GRCh38 were obtained from the SPRINT website. Predicted RNA editing events were primarily AG on the plus strand or TC on the minus strand, consistent with A-to-I editing expectations. Non-AG/TC events were excluded from further analysis, and only events overlapping annotated Alu repetitive elements were retained. To ensure specificity for the RNA editing events, detected loci were filtered against known human SNPs using bedtools intersect [34] with the dbSNP dataset (00-common_all.vcf; NCBI). Events overlapping TC or AG SNPs were removed, resulting in the exclusion of 1.02% of the identified sites. The final dataset, in BED format, included the total number of reads mapped to each locus and the number of reads supporting each RNA editing event (available at http://rhesus.amu.edu.pl/share/UGGT1-AS1_SupplData/ (accessed on 1 April 2025) and http://yeti.amu.edu.pl/UGGT1-AS1_SupplData/ (accessed on 1 April 2025)).

### 4.3. Cell Culturing

MCF7 cells, provided by a ATCC supplier (ATCC, Manassas, VA, USA), were cultured in RPMI medium (Capricorn Scientific, Ebsdorfergrund, Germany) with 10% fetal bovine serum (FBS) (Capricorn Scientific, Ebsdorfergrund, Germany) and Antibiotic–Antimycotic (100×) (Thermo Scientific™, Waltham, MA, USA). The cells were maintained in an incubator at a temperature of 37 °C and an atmosphere of 5% CO2.

### 4.4. Subcellular Fractionation

Subcellular fractionation was carried out as previously described [35]. The method was based on differential centrifugation in buffers. Cells were fractionated for RNA isolation purposes and divided into cytoplasmic, nuclear, chromatin and nucleoplasmic fractions. RNA was isolated using TRIreagent (Molecular Research Center, Cincinnati, OH, USA), reverse transcribed with a RevertAid RT Reverse Transcription Kit (Thermo Scientific™, Waltham, MA, USA) and used to perform a real-time qPCR experiment.

### 4.5. DNA and RNA Isolation

Total RNA and DNA were isolated from cell lines using TRIreagent (Molecular Research Center, Cincinnati, OH, USA) following manufacturer’s protocol. In order to check quality and quantity of nuclear acids, a spectrophotometer (DeNovix, Wilmington, DE, USA) was used. To make sure that parameters of isolated RNA were meeting criteria of ratios 260/280 and 260/230 to be approximately 2.0, an RNA Clean & Concentrator™ (ZYMO Research, Irvine, CA, USA) clean-up kit with additional step of DNase I Treatment was used.

### 4.6. RT-PCR and Sanger Sequencing

RNA was reverse transcribed to cDNA with a RevertAid First Strand cDNA Synthesis Kit (Thermo Scientific, Waltham, MA, USA) according to the manufacturer’s protocols. cDNA was used for polymerase chain reaction (PCR) with the high-fidelity polymerase EconoTaq PLUS2X Master Mix (Lucigen, Middleton, WI, USA). The primers were designed using the PrimerBlast program to specifically target the gene of interest, ensuring similar melting temperatures and an adequate product size (e.g., <200 bp for qPCR). All primer sequences used for this study are specified in Supplementary File S1. The reactions were prepared in a total volume of 10 μL, and half of the product was used for electrophoresis in a 1.5% agarose gel containing GelRed (Biotium, Fremont, CA, USA) in a 1X TAE buffer. Photographs of the gels were taken using G: Box EF2 (Syngene, Cambridge, UK) with GeneSys image analysis software version 1.6.1.0 (Syngene, Cambridge, UK). The remaining 5 μL of the PCR product was used for Sanger sequencing after cleaning with ExoSAP-IT™ PCR Product Clean-up (Thermo Scientific, Waltham, MA, USA). The results of the Sanger sequencing were analyzed using Chromas software version 2.6.6.

### 4.7. Quantitative Real-Time PCR

Reverse transcribed RNA and primers were obtained in the same manner as in the PCR. Quantitative real-time PCR was performed on a QuantStudio™ 7 Flex Real-Time PCR System platform (Thermo Scientific, Waltham, MA, USA) using PowerUp™ SYBR Green Master Mix (Thermo Scientific, Waltham, MA, USA) according to the manufacturer’s protocols. Three biological and three technical replicates were carried out, and the calculations of relative gene expression were performed using the 2^−ΔΔCT^ method [36].

### 4.8. Gene Silencing with LNA GapmeRs

Antisense LNA GapmeRs (Qiagen, Hilden, Germany) were designed using the Qiagen online tool to uniquely target transcripts of *UGGT1-AS1* while ensuring no off-targets on its sense partner due to their strand-specific nature. Additionally, negative control GapmeRs (which have no affinity to any known human lncRNA or mRNA sequences) and positive control GapmeRs, targeting *MALAT1*, were applied. Transfection was performed for 72 h using an Opti-MEM (Thermo Scientific, Waltham, MA, USA) cell medium with a lipofectamine 3000 reagent (Thermo Scientific, Waltham, MA, USA). The experiment was performed and optimized for an MCF7 cell line. The effectiveness of silencing was checked with RT-qPCRs.

### 4.9. MTT Assay

Cells were transfected with two different LNA GapmeRs, one targeting *UGGT1-AS1* and one used as a negative control. They were then seeded on 96-well plates (5000 cells per well) and treated with 1 mg/mL tetrazolium substrate (3-(4,5-dimethylthiazol-2-yl)-2,5-diphenyltetrazolium bromide) MTT (Merck Millipore, Burlington, MA, USA). After 4 h incubation, formazan crystals were dissolved in 200 μL DMSO. Absorbance was measured at OD = 570 nm with a Tecan Spark^®^ Multimode Microplate Reader (Tecan Group, Männedorf, Switzerland).

### 4.10. RNA Stability Assay

RNA stability was checked using actinomycin D (Thermo Scientific, Waltham, MA, USA). First, transfection with LNA GapmeRs was performed, as described earlier. A total of 72 h after transfection, actinomycin D was added to the cells to the final concentration of 1 mg/mL. Cells were collected at different timepoints (0 h, 0.5 h, 1 h, 2 h, 4 h, 6 h and 8 h after adding actinomycin D) and RNA was extracted to perform RT-qPCR.

### 4.11. RNA Antisense Purification (RAP-RNA)

The RAP-RNA method was performed according to the Guttman Lab protocol [37]. Cell crosslinking was carried out in Spectrolinker at a 350 nm wavelength to preserve endogenous RNA complexes. A key feature of RAP is its use of long (>60-nt) capture probes, enabling hybridization and wash conditions that minimize nonspecific interactions. Biotinylated probes (Supplementary File S1) were designed in PrimerBlast and purchased from Merck (Merck Millipore, Burlington, NJ, USA). to specifically bind transcripts of interest. Fixed endogenous RNA:RNA complexes were purified by hybrid capture with probes, then streptavidin C1 magnetic beads (Thermo Scientific, Waltham, MA, USA) were used for magnetic separation and isolation of endogenous complexes. An important step of this procedure was adding an RNase H enzyme that selectively digests ssDNA-RNA hybrids to ensure that only sense transcripts were left. The final RNA was purified with SILANE beads (Invitrogen, Waltham, MA, USA) and used for RT-PCR.

### 4.12. Statistical Analysis

Statistical analyses were performed using GraphPad Prism version 10.4.1 for Windows (GraphPad Software, www.graphpad.com (accessed on 1 April 2025)). For in vitro experiments, statistical comparisons were made with *t*-test or ANOVA. *p*  <  0.05 was considered statistically significant. All data represent the mean ± standard deviation (SD) from at least three replicates.

## 5. Conclusions

In conclusion, our findings highlight the important role of *UGGT1-AS1* in modulating *UGGT1* expression, with potential implications for cancer biology. As NATs continue to emerge as key regulatory molecules, further research into their *UGGT1-AS1* mechanisms may uncover novel therapeutic strategies for targeting cancer and other diseases.

## Data Availability

The datasets generated and/or analyzed during the current study are available at http://rhesus.amu.edu.pl/share/UGGT1-AS1_SupplData/ (accessed on 1 April 2025) and http://yeti.amu.edu.pl/UGGT1-AS1_SupplData/ (accessed on 1 April 2025).

## Supplementary Materials

The following supporting information can be downloaded at https://www.mdpi.com/article/10.3390/ijms26115108/s1.
