# Supplementary material for: Novel lncRNA UGGT1-AS1 Regulates UGGT1 Expression in Breast Cancer Cell Line
Source: Int J Mol Sci. 2025 May 26;26(11):5108. doi: 10.3390/ijms26115108 (PMC12155537; doi:10.3390/ijms26115108)
Supplement: Supplementary file 1 [file ijms-26-05108-s001.zip › ijms-3601524-supplementary.pdf]

A novel lncRNA *UGGT1-AS1* regulates *UGGT1* expression in breast cancer cell line

Klaudia Samorowska[1], Elżbieta Wanowska[1], Michał Wojciech Szcześniak[1]

[1] Faculty of Biology, Adam Mickiewicz University in Poznan, Institute of Human Biology and Evolution, Uniwersytetu Poznańskiego 6, Poznań, Poland

Supplementary File S1. A list of primers used for PCR and qPCR reactions, Sanger sequencing as well as biotinylated probes used for RNA antisense purification (RAP) and *UGGT1-AS1* GapmeR sequence.

Oligo name	Sequence
**UGGT1-AS1_FOR_115bp**	TCCAAGTCACGGGGTCAGTA
**UGGT1-AS1_REV_115bp**	TAGAGGGCCAAGAGCTAGGG
**UGGT1_ qPCR_FOR**	AGATGCTTGCCTGTCTGCTT
**UGGT1_ qPCR_REV**	TCTCTATCTCTTGACCTCATAA
**UGGT1-AS1_FOR_184bp**	TGGGCCAGTCTCTTCCCCCT
**UGGT1-AS1_REV_184bp**	CATGATCCACCTGCCTCGGCCTCC
**ACTB_FOR**	TTAAGGAGAAGCTGTGCTACGTC
**ACTB_REV**	AGTTTCGTGGATGCCACAGG
**GAPDH_FOR**	GATGACAAGCTTCCCGTTCTC
**GAPDH_REV**	TGAAGGTCGGAGTCAACGGA
**H19_FOR**	ATCGGTGCCTCAGCGTTCGG
**H19_REV**	CTGTCCTCGCCGTCACACCG
**MALAT1_FOR**	TTTTAGCAACGCAGAAGCCC
**MALAT1_REV**	ATACCACCACCTGGAATGGC
**1UGGT1-AS1_RAP**	[Btn]TATGTCCGGTCAAGCCCTCGTGTTCTGAACCTTGTCAA GGCTCTGCCTGAATGAGTTTTCATCTGATTGTGGGGACCAA GTCCCTGAGTAGAGGGCCAAGAGCTAGGGACAGGGGGAAG
**2UGGT1-AS1_RAP**	[Btn]AGACTGGCCCAGGTGGTAGGGAGGAAAGAACTCCCAG AGTTTCCTTTAGCCAGGAAACCTGCTCTACTGACCCCGTGA CTTGGACAGTCAGACATCACCCTGAGAGTGACAAGTGTAA
**3UGGT1-AS1_RAP**	[Btn]AATGACTCCCTTCCTCCCCCGCCCTCCGGAAGTATATTT AGATACTTGAAAGCAGTCCTTTTCTAAAATGGCCTTACCTAT GTGGCCTGAACGATTAAAAGAAAGAACTCAG
**UGGT1-AS1_GapmeR**	AAGTGTAA AATG ACTC
